# Can Bacterial Manipulation Deliver Reef-Scale Thermal Enhancement of Corals?

**DOI:** 10.3390/microorganisms14010202

**Published:** 2026-01-15

**Authors:** Madeleine J. H. van Oppen, Talisa Doering, Luanny Martins Fernandes

**Affiliations:** 1 Australian Institute of Marine Science, Townsville, QLD 4810, Australia; 2 School of BioSciences, The University of Melbourne, Parkville, VIC 3010, Australia; t.doering@unimelb.edu.au (T.D.); luanny.martinsfernandes@student.unimelb.edu.au (L.M.F.)

**Keywords:** probiotics, microbiome manipulation, assisted evolution, coral reefs, coral bleaching, climate change

## Abstract

A rapid decline of coral reefs is taking place around the world, with climate warming being the biggest driver behind this deterioration. Efforts to increase coral climate resilience via bioengineering methods have thus become urgent, and there is hope that such interventions can help corals and coral reefs survive until a time when no further climate warming occurs and perhaps a future of climate cooling is imaginable. The manipulation of coral-associated bacterial communities is among the less advanced interventions currently being explored. Nevertheless, early findings provide confidence that some level of thermal enhancement can be achieved via the inoculation of corals with beneficial bacteria. The small number of studies available, however, is limited in terms of the traits used to select candidate bacteria and their ability to ascribe host enhancement to specific bacterial taxa and functions. Further, findings to date are unable to decipher whether candidate bacteria integrate stably within the coral microbiome. These shortcomings prevent assessment of the efficacy of bacterial manipulation to enhance the long-term thermal resilience of corals on the reef. Here we summarise the state-of-play of the field and provide recommendations to fast-track this approach via fine-tuning experimental designs and methods.

## 1. Introduction

Most stony corals (Scleractinia) harbour dinoflagellate microalgae in the family Symbiodiniaceae on which they rely for the bulk of their nutrition, and they cannot live without these photosymbionts for prolonged periods of time [1]. Alarmingly, the loss of the photosymbionts from the coral tissues, i.e., coral bleaching, and resulting coral mortality have become regular phenomena on coral reefs around the world [2,3]. While coral bleaching can be caused by various stressors, the combination of high temperature and high irradiance that occurs during summer heatwaves is the primary cause of coral bleaching and the only cause of mass coral bleaching [4]. Summer heatwaves have increased in frequency and severity due to climate change, with the most extreme thermal anomalies recorded during the 2023–2025 global mass bleaching event [5,6,7] when almost 80% of the world’s coral reefs bleached [8]. Coral cover worldwide has declined by an estimated 50% over the past 30–50 years [2,9]. Climate models predict further intensification of summer heatwaves into the future and additional loss of coral reefs [10,11]. While natural adaptation of corals to rising temperatures is occurring [12,13] and further adaptation is possible, the natural rate of adaptation is likely too slow to keep up with rapid climate warming [14,15]. In support of this notion, a simulation of eco-evolutionary dynamics of coral populations across 3800 reefs on the Great Barrier Reef forecasts a rapid decrease in coral cover over the next two decades, from 38.8% in 2023 to 17% by 2040, irrespective of the emission scenario [15]. Therefore, human interventions are required to assist coral adaptation to climate change and the persistence of coral reefs into the future [16,17,18]. Here we synthesise research on one such intervention, i.e., the manipulation of coral-associated bacteria that aims to enhance coral thermal tolerance; we highlight the strengths and weaknesses of common experimental designs and provide recommendations for a more targeted exploration of the efficacy of this intervention for implementation at an ecologically relevant scale on the reef.

## 2. Manipulating Host-Associated Microbes for Biodiversity Conservation

Plants and animals are holobionts, unable to function and survive without microbial symbionts. Microorganisms influence the physiology of their plant and animal hosts, and tweaking the host-associated microbiome may alter the holobiont phenotype [19]. Microbiome manipulation may involve transplantation of whole microbiomes from one individual (typically healthy) to another (typically impaired or diseased), as in faecal microbiota transplantation (FMT) where a stool sample is transplanted from a healthy colon into a diseased colon; this procedure is often successful in curing patients from *Clostridioides difficile* infection [20]. Alternatively, the host can be inoculated with one or a few live microbes (probiotics). Probiotics are frequently used with the aim of improving human health (often orally administered), but they are also applied in other settings such as in the aquaculture industry and as wildlife medicine. For example, augmenting the amphibian skin microbiome with certain bacteria can sometimes offer protection against infection with the chytrid fungus *Batrachochytrium dendrobatidis* [21]. Probiotic microorganisms are not always stably incorporated into the host microbiome and repeated inoculations may be required to maintain the benefits they provide [22]. Repeated inoculations are feasible in the context of human health and farming plants and animals in contained facilities, but are less achievable for wild populations. The stable introduction of a single microbial strain into a host organism is another intervention able to induce phenotypic alterations. A well-known example is the introduction of *Wolbachia* bacteria into *Aedes* mosquitoes, which induces reproductive incompatibility when infected male mosquitoes inseminate females lacking *Wolbachia* or harbour a different *Wolbachia* strain. Further, certain *Wolbachia* strains reduce vector competence of dengue-infected mosquitoes [23].

Infection with mutualistic microbes may also increase host fitness under heat stress [24]. For instance, thermotolerance of the whitefly, *Benisia tabaci*, was enhanced by infection with *Rickettsia* bacteria. *Rickettsia* induces constitutive expression of host cytoskeleton and microfilament genes, which act in concert with other genes such as heat shock protein (HSPs) genes to increase thermal tolerance [25]. A symbiont-induced host gene ‘front-loading’ mechanism has also been observed in corals inoculated with experimentally evolved photosymbionts (*Cladocopium proliferum* in the family Symbiodiniaceae). These coral hosts exhibited constitutively higher transcript abundance for HSPs and other chaperones, glutathione peroxidase, glutathione synthase, glutathione S-transferases, and superoxide dismutases, among others, as well as enhanced thermal tolerance relative to corals with wildtype symbionts [26]. In wheat and *Arabidopsis*, infection with a root endophyte (the bacterium *Enterobacter* SA187) induced sustained methylation in several host genes, driving their expression and boosting thermal tolerance [27]. Further, treatment of the worm, *Caenorhabditis elegans*, with the probiotic bacterium *Lactobacillus gasseri* SBT2055 upregulated a transcription factor and its target genes that induced an antioxidant response and prevented reduction in mitochondrial function under stress conditions [28]. Most microbial manipulation studies have targeted host-associated bacteria, and the successes of bacterial manipulation seen in many organisms and biological systems suggest there is potential for this intervention in corals also.

## 3. Bacterial Communities of Scleractinian Corals

### 3.1. Taxonomic Diversity and Affiliation

Coral-associated bacterial communities exhibit a vast diversity, with some coral species or individuals harbouring high-diversity communities of hundreds to thousands of bacterial taxa [29], while others comprise low-diversity communities containing fewer than one hundred strains [30]. The latter mostly consist of cell-associated microbial aggregates (CAMAs) within coral tissues, which are composed of various Endozoicomonadaceae strains (mostly in the genus *Endozoicomonas*) [30,31,32,33,34]. Occasionally, CAMAs comprise *Simkania* (Simkaniaceae family) [34], *Soroendozoicomonas* (Endozoicomonadaceae) [35] and *Aquarickettsia* (Midichloriaceae, referred to as ‘Rickettsiales-like organism clusters (RLOs)’) [36]. The most dominant taxa found in coral bacterial communities are Proteobacteria, Firmicutes, Bacteroidetes and Cyanobacteria [37]. Among these, the bacterial genera *Endozoicomonas*, *Ruegeria*, and *Vibrio* are both the most abundant and widely distributed across coral species. At the bacterial family level, this trend is reflected in the high abundance and broad distribution of Rhodobacteraceae.

### 3.2. Bacterial Occurrence Across Coral Microhabitats

**Figure 1:**
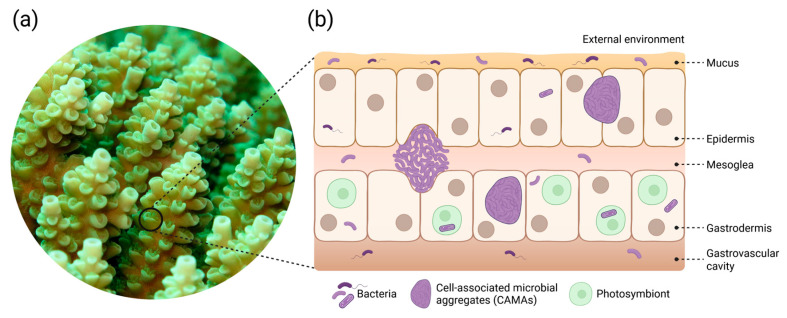
Overview of coral tissue organisation and bacterial occurrence across coral microhabitats. (**a**) Photograph of a section of an *Acropora* colony showing polyps with tentacles inside the corallites (the cuplike calcium carbonate skeleton of a single polyp). (**b**) Schematic cross-section of the oral coral tissue and the microhabitats in which photosymbionts and bacteria reside. Bacteria occur in the mucus, epidermis, mesoglea, gastrodermis, gastrovascular cavity, skeleton (not shown in this schematic), and within the photosymbionts. Tissue-associated bacteria sometimes form aggregates called cell-associated microbial aggregates (CAMAs). CAMAs can occur in the epidermis, gastrodermis and mesoglea and can be intra- or extracellular. Photograph by Justin Maire and Ashley Dungan. Created in BioRender. Martins Fernandes, L. (2026) https://BioRender.com/f2z9sf6 (accessed on 7 January 2026).

Bacteria occur across all coral microhabitats (Figure 1), i.e., the mucus [38], tissue [34,39], skeleton [40] and gastrovascular cavity [41]. Recently, bacteria have also been localised in the mesoglea within coral tissues, a habitat long thought to be void of bacteria [42]. Further, coral photosymbionts host intracellular bacteria [43]. Of all coral microhabitats, the coral mucus layer is believed to host the most variable and transient assemblages of bacteria [44], while bacteria associated with coral tissues may be more stably associated [45,46,47]. In terms of overall abundances, corals host a greater proportion of transient bacteria relative to stable bacterial symbionts [48]. For coral microbiome manipulations, there may be value in focusing on the more stable tissue-associated bacteria to increase the likelihood of having long-term benefits that can assist corals to withstand summer heatwaves over multiple years and possibly throughout their lives.

### 3.3. Functions of Coral-Associated Bacteria

Bacteria are important players in coral health and fitness through a wide range of functions, including the production of antimicrobial compounds and antioxidants, or by cycling nutrients such as nitrogen, sulfur, carbon and phosphorus [49]. Bacteria are therefore thought to play important roles in host defence, particularly against pathogens. Some *Vibrio* species are directly implicated in the development of bleaching, whereas other diseases, such as black band disease, involve complex consortia of microbes [50,51].

The coral probiotic hypothesis poses that coral microbiome communities are shaped through a dynamic interaction between the host and the environment, leading to a beneficial bacterial community that contributes to the ecological success of the coral host [52]. However, definitive evidence supporting or refuting the hypothesis is still lacking, as the fitness benefits of microbiome shifts to the host remain unclear. Changes in the microbiome composition may be the result of “shuffling”, i.e., abundance shifts in bacterial taxa that are already present within the coral microbiome or “switching”, i.e., the acquisition of new taxa by the holobiont [53]. Coral microbiomes have been reported to sometimes change in their composition in response to different environmental pressures such as pollution or ocean warming [46], and the composition of coral-associated bacterial communities can be correlated with heat tolerance of the host [54,55]. This suggests that bacteria and their functions could fine-tune the holobiont’s physiological response to heat stress. Changes in bacterial composition may also signal dysbiosis, where homeostasis breaks down and opportunistic pathogens proliferate [56]. One common feature of microbiome dysbiosis is an increase in beta diversity, reflecting higher dissimilarity among microbial community composition [57]. Overall, defining healthy versus diseased or disturbed coral microbiomes is challenging due to the spatial and temporal complexity of these host-associated communities [58].

### 3.4. Temporal Changes in Coral-Associated Bacterial Community Composition

Another layer of complexity is added by the change in coral-associated bacterial communities with host developmental stage and age [46]. Coral early life stages are better able to establish new associations with bacteria from the environment [59] compared to adult corals that have undergone selection processes creating a more stable bacterial community [60,61]. Despite hosting less variable communities, adult corals exhibit varying degrees of microbiome flexibility in response to environmental changes, depending on the coral species and the composition of their established bacterial community [62]. When cross-transplanted between reef sites with differing levels of anthropogenic impact in the Red Sea, *Acropora hemprichii* colonies with high-diversity microbiomes containing several equally abundant bacterial taxa shifted their bacterial community composition. In contrast, the low-diversity microbiomes of *Pocillopora verrucosa*, dominated by *Endozoicomonas* spp., remained largely unchanged after transplantation. These findings highlight that different coral species may have different degrees of microbiome flexibility, which could represent distinct adaptative strategies [63]. An important open question remains as to how microbiome flexibility relates to coral host resilience under repeated stress events, such as thermal stress, because no studies have yet examined microbiome dynamics under repeated summer heatwaves. Overall, harbouring a flexible community is a double-edged sword; while it may aid holobiont acclimatisation to environmental changes, it may also come at the cost of losing previously essential symbiotic partners [58,63]. Note that not all shifts in community composition might necessarily affect coral holobiont functioning. Some changes may be neutral if incoming bacteria replace the functions of those that are lost, or if the displaced taxa were functionally redundant. In some cases, the mere presence of certain bacteria may have little relevance for host fitness. In contrast, a less flexible microbiome may confer protection through the maintenance of beneficial bacteria across repeated thermal stress events.

When attempting to manipulate coral-associated bacterial communities, it is essential to account for the substantial variability in these communities across time, environmental conditions, spatial scales, developmental stages, host health states, coral species, and the structure of the existing microbiome. Together, these coral microbiome characteristics highlight the considerable complexity involved in manipulating coral bacterial communities.

## 4. State-of-Play of Bacterial Probiotics Aimed at Enhancing Coral Thermal Tolerance

Correlations between the community composition of coral-associated bacteria and coral thermal tolerance suggest bacteria can influence the upper thermal tolerance limit of corals [54,55]. This observation has instigated research into the feasibility of bacterial manipulations targeted at helping corals to withstand or recover from thermal stress. Doering et al. [64] showed that transplantation of whole microbiomes from thermally tolerant to conspecific sensitive colonies can enhance tolerance to subsequent thermal stress. The downsides of using whole microbiome transplants for corals, however, are that (1) donor corals or coral fragments need to be sacrificed to obtain the microbiomes, which may not be justifiable on depauperate reefs and which may also be logistically challenging, (2) microbiomes vary over time and space and it is unlikely one will be able to obtain identical or highly similar microbiomes from independent isolations, (3) there is a risk that pathogens will also be transplanted. We therefore do not recommend the use of whole microbiome transplants for enhancing coral thermal tolerance long-term at the reef scale. Further, six laboratory-based studies using cultured, potentially beneficial bacteria (i.e., candidate bacteria) have been carried out to date, and these have concluded that inoculating corals with candidate bacteria increases the fitness of corals during or following heat stress. Experimental parameters in these investigations varied (Table 1), making it difficult to compare them. Candidates were typically selected based on their culturability from coral hosts or surrounding seawater, followed by screening for functions considered beneficial to the coral under thermal stress (see Section 5). However, most of these functional assays were limited to a small number of tests that may not reflect bacterial activity within the coral host. Similarly, the assessment of the host stress response was mostly based on a small number of traits and methods for quantifying traits were in some cases substandard. For instance, coral colour rather than actual photosymbiont cell counts was commonly used [65,66,67,68], and in some of the cited studies no statistical analysis of colour was conducted. For coral species with patchy photosymbiont distribution across the polyps and colony, such as the commonly used *Pocillopora damicornis*, this method can be inaccurate. Across several studies [65,67,69] the same bacterial strains were repeatedly employed for inoculation. Other studies used different strains from various coral species but screened for similar functions [66,70]. De Breuyn et al. [71] used strains from five coral species and reported that these were screened for beneficial functions but did not specify which strains exhibited which traits. Changes to the resident microbiome community were observed in some studies (Table 1), but the taxa that changed in relative abundance were not consistent from study to study. It remains unresolved whether microbiome restructuring following administration of candidate bacteria is beneficial to coral fitness. While this is often interpreted as such in the coral probiotic studies cited, there is currently no evidence to support it. A better understanding of microbiome changes and factors that drive them is required.

Field inoculation of corals has so far occurred in only one study [72]. A bacterial consortium comprising six strains was administered three times per week over a 3-month period, and the inoculated as well as the no-inoculum control corals were subsequently surveyed for five months. No treatment effects on the in situ coral phenotypes were observed, nor did bacterial inoculation enhance thermal tolerance as assessed in the laboratory by a rapid heat stress assay (18 h) four times throughout the experiment. Changes to the resident microbiome composition were only seen at the end of the inoculation period (i.e., not before, during, or five months after the last inoculation), at the time when three of the six genera to which the candidate bacteria belonged showed slightly (but statistically significant) higher relative abundances in the inoculated corals.

Long-term thermal tolerance enhancement (i.e., more than a few months) following inoculation of corals with candidate bacteria has never been tested in any of the laboratory studies conducted to date (Table 1; the duration of all laboratory-based studies has been less than 45 days). The field-based study by Delgadillo-Ordoñez et al. [72] was run for ~8 months but found no evidence of improved thermal tolerance as a consequence of bacterial inoculation. Therefore, it is currently unknown whether sustained thermal enhancement can be achieved via bacterial manipulation.

**Table 1:** Summary of published laboratory studies where corals were inoculated with cultured bacteria and exposed to ambient and/or elevated temperature. Dpi = days post inoculation.

Coral Species	Number of Inoculations	Number of Bacterial Strains in Inoculum	Duration of Experiment	Dpi when a Candidate Was Detected *	Relative Abundance of Candidates at Elevated Treatment % *	Inoculation Caused Changes in Resident Microbiome	Inoculation Enhanced Fitness	Reference
Studies aiming to enhance coral thermal resilience via inoculation with bacteria								
*Pocillopora damicornis*	2	7	10 d acclimation, 26 d elevated temperature	9	0–0.7	Y	Y	[65]
*Mussimilia hispida*	11	6	30 d acclimation, 26 d elevated temperature, 19 d recovery	4 ^a^	0–0.25	Y ^b^	Y ^c^	[66]
*Pocillopora damicornis*	2	7	18 d acclimation, 29 d elevated temperature	Not assessed	Not assessed	Not assessed	Not assessed ^d^	[69]
*Pocillopora damicornis*	7	7	18 d acclimation, 34 d elevated temperature	6 ^e^	0–0.2	N	Y	[67]
*Pocillopora damicornis*	1	1	7 d acclimation, 28 d elevated temperature	Results inconclusive ^f^	Results inconclusive ^f^	Y	Y	[68]
*Acropora* cf. *hemprichii*, *Pocillopora verruscosa*	1	4 ^g^	2 hr acclimation, 48 h elevated temperature	Not assessed	Not assessed	Not assessed	Y	[71]
Studies examining effect of bacterial inoculant or its temporal stability at ambient temperature								
*Pocillopora damicornis*	3	4	7 d acclimation plus an additional 21 d at ambient temperature	Not detected	0	Y	Y ^h^	[70]
*Acropora carduus*	1	1	7 d acclimation plus an additional 6 d at ambient temperature	Not assessed	Not assessed	Y	Y/N ^i^	[73]
*Acropora kenti*	4	1 ^j^	17 d	5	0.59–57	Y	Not assessed	[74]

* ASVs of strains in the inoculum were shown to be or were likely identical to resident bacteria based on 16S rRNA gene metabarcoding, meaning that their presence and relative abundance have considerable uncertainty also see note ^e^. ^a^ No candidate bacteria were detected four days after the last inoculation during the recovery phase, only during the heat stress phase. ^b^ Only during the heat stress phase. ^c^ Enhancement was mostly seen during the recovery phase, and not as much during exposure to heat stress. ^d^ Skeletal mass of coral branch tips and the amount of calcium on the skeletal surfaces of the corals were higher in inoculated compared with no-inoculum control corals at elevated temperature. ^e^ One candidate was detected prior to the first inoculation, meaning some resident strains are identical in the 16S rRNA V3-V4 region Figure 2a in Cardoso et al. [67]. ^f^ The administered bacterium was only detected at the last sampling time point 14 days after inoculation. However, it was detected in all treatments, including in corals that were not inoculated. ^g^ Only the *Acropora hemprichii* experiment is presented, because *Pocillopora verrucosa* showed no signs of stress in response to the elevated temperature profile the corals were subjected to. ^h^ Energy reserves and growth rate. ^i^ Inoculation with *Ruegeria profundi* prevented *Vibrio coralliilyticus*-induced bleaching and polyp tissue loss. In the absence of *V. coralliilyticus*, no fitness benefits of the *R. profundi* inoculation were recorded. ^j^ Eight strains were tested in total, each in a separate treatment. Therefore, each inoculum contained only one strain.

**Figure 2:**
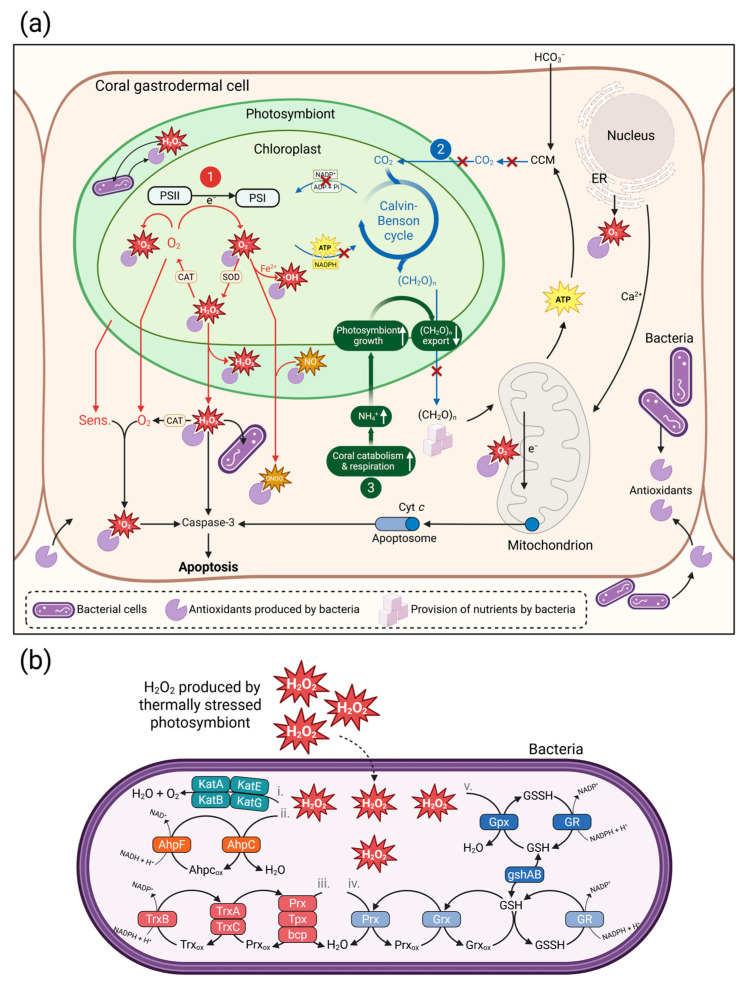
Cellular mechanisms of coral bleaching and bacterial traits proposed to enhance coral thermal tolerance. (**a**) There are currently three coral bleaching theories, which are indicated by numbers 1–3 in the figure and described in Section 6.1. ADP = adenosine diphosphate, ATP = adenosine triphosphate, Pi = inorganic phosphate, CCM = carbon concentrating mechanism, Cyt *c* = cytochrome c, ER = endoplasmic reticulum, NADP^+^ = nicotinamide adenine dinucleotide phosphate, NADPH = nicotinamide adenine dinucleotide phosphate (reduced), PSI and PSII = photosystem I and II, CAT = catalase, SOD = superoxide dismutase, Sens. = O_2_-sensitising metabolites. Hypothesised mechanisms by which bacteria may enhance coral thermal resilience, such as extracellular enzymatic and non-enzymatic antioxidant production and nutrient provisioning, are indicated by the purple icons displayed in the legend. (**b**) When excess H_2_O_2_ is produced by thermally stressed photosymbionts, it can diffuse across cellular membranes into associated bacterial cells, where several intracellular antioxidant pathways may act to scavenge it. These potential pathways are described in Section 5.1. Created in BioRender. Martins Fernandes, L. (2026) https://BioRender.com/90flqhz (accessed on 7 January 2026).

## 5. Bacterial Traits Previously Proposed to Directly Enhance Coral Thermal Resilience

Thermal tolerance enhancements of the coral host following treatment with a probiotic may be caused by the candidate bacteria providing direct benefits. Possible beneficial traits that have been proposed in previous research are antioxidant capacity, carbon-provisioning, nitrogen fixation and cycling, mycosporine-like amino acid production, iron acquisition via siderophores, phosphate solubilising activity, and urease activity. Each of these traits is addressed below.

### 5.1. Antioxidant Capacity

An excess production of reactive oxygen species (ROS) within the coral holobiont during thermal stress is predicted by current bleaching theories [75,76,77,78]; see Figure 2a and Section 6.1) and ample evidence exists to support this [79]. Coral-associated bacteria have been hypothesised to scavenge these ROS through various mechanisms, potentially mitigating the bleaching cascade at multiple stages (Figure 2a). High antioxidant capacity, particularly the neutralisation of H_2_O_2_ via enzymatic antioxidants including catalases and peroxidases, is believed to be an important trait of probiotic candidate bacteria [80,81]. H_2_O_2_ can pass biological membranes and is able to exit photosymbiont cells and enter the bacterial cytosol, where it can be scavenged by antioxidant enzymes produced by the bacterium (Figure 2b). These may include (i) calatases (KatA, KatB, KatE, and KatG), which directly decompose H_2_O_2_ into water and oxygen in a single-step reaction; (ii) the alkyl hydroperoxide reductase system (AhpC-AhpF), via which AhpC reduces H_2_O_2_ and becomes oxidised (AhpC_ox_), and is then reactivated by AhpF using NADH as electron donor; (iii) thioredoxin-dependent peroxiredoxins (Prx/Tpx/bcp), which are oxidised (Prx_ox_) while reducing H_2_O_2_ and are restored to their active form by thioredoxins (TrxA/TrxC) that are regenerated by thioredoxin reductase (TrxB); (iv) glutaredoxin-dependent peroxiredoxins, in which Prx_ox_ is recycled by glutaredoxin (Grx) using glutathione (GSH) as the electron donor, and its subsequent reduction by glutathione reductase (GR); and (v) the glutathione peroxidase (Gpx) system, in which Gpx reduces H_2_O_2_ to water by oxidising GSH to GSSG, with GSSG being restored to GSH by GR using NADPH. The intracellular GSH pool required for these pathways is maintained through de novo glutathione synthesis encoded by the gshAB operon (Figure 2b). Additionally, some bacteria produce extracellular antioxidants [82,83]; Figure 2a). Non-enzymatic antioxidants such as carotenoids, dimethylsulfide (DMS) and its main precursors, dimethylsulfoniopropionate (DMSP) and dimethylsulfoxide (DMSO), are scavengers of other ROS [84]. DMSP can be degraded via demethylation by the gene *dmdA*, and the presence of only this gene has previously been used as a criterion for choosing bacterial candidates in corals. Corals generally have very high concentrations of DMSP and its breakdown products; therefore, these sulfur compounds are deemed important in holobiont functioning [85]. Most bacterial manipulation studies on corals carried out so far have considered antioxidant traits to select candidate bacteria, but these have been limited to a few individual functions from the broader range of bacterial ROS-scavenging mechanisms.

### 5.2. Carbon-Provisioning

Host starvation is hypothesised to be the cause and/or outcome of coral bleaching. In support of this notion, numerous studies have shown that fed corals are more bleaching-tolerant than unfed corals, and in the wild, corals exhibiting greater heterotrophy are more thermotolerant [86,87]. Therefore, bacteria that can provide the host with nutrients, particularly carbon-based nutrients, may represent good candidates to mitigate bleaching (Figure 2a). Such bacteria would have to be autotrophic, such as photoautotrophic cyanobacteria [37] that use light as an energy source, or chemolithoautotrophs that use an inorganic electron donor such as hydrogen sulfide (H_2_S) or ammonium (NH_4_^+^) [88]. The ability to export sugars would be important, given that photosynthate produced by the photosymbionts is primarily exported to the coral host in the form of sugars (mostly glucose). Some bacteria have the semiSWEET gene, a homologue of the eukaryotic SWEET (sugars will eventually be exported transporter) which encodes a protein that exports glucose [89]; while the function of semiSWEET has not been validated, it has been speculated bacteria also use it to export glucose [81]. To date, studies aiming to enhance coral thermal tolerance have not tested candidates that were screened for this trait.

### 5.3. Nitrogen Fixation and Cycling

Some studies argue bacteria that fix dinitrogen (i.e., have the *nifH* gene) or have the gene for the reduction of nitrite (NO_2_^−^) to nitric oxide (NO) (denitrification) are beneficial to corals during thermal stress [65,66,67,70]. However, while N_2_-fixation by diazotrophic bacteria increases under elevated temperature, the additional ammonium is not assimilated by the photosymbionts or the coral host [90], and it unclear why this function has been suggested as beneficial to corals that experience heat stress. The benefits of the reduction of nitrite (NO_2_^−^) to nitric oxide (NO) for the coral are also nebulous. However, other nitrogen cycling enzymes such as those able to complete the nitrification process of ammonium to nitrate (i.e., ammonia oxidation plus nitrite oxidation, or complete nitrification by comammox, such as *Nitrospira* bacteria) could be beneficial during thermal stress as this would lower ammonium concentrations within the host; at the same time, these bacteria fix CO_2_ with the energy (electrons) released by these oxidation processes, which may be used for their own growth and possibly supply the coral with carbon compounds. The resulting nitrate could still be assimilated by the photosymbionts [91], but it is more energy-costly than assimilating ammonium as it involves the nitrate reduction step. To date, bacterial manipulation studies have only tested candidate bacteria possessing the genes for nitrogen fixation or for the reduction of nitrite to nitric oxide, but no studies have assessed bacterial candidates capable of completing the full nitrification process from ammonium to nitrate.

### 5.4. Mycosporine-like Amino Acid Production

Mycosporine-like amino acids can protect against UV-radiation [92], which can be high during summer heatwaves, potentially causing additional stress. So far, no candidate bacteria tested for this trait have been used.

### 5.5. Iron Acquisition via Siderophores

Siderophores bind ferric iron, providing increased bioavailability of iron to the photosymbionts [92]. Marine bacteria use siderophores to chelate iron in their surrounding environment and transport it into their cells. Microbes, including dinoflagellates [93], can sometimes take up siderophores produced by another species [94]. If coral photosymbionts are able to take up siderophores produced by holobiont-associated bacteria, nitrate would become a more available nitrogen source because nitrate assimilation enzymes require a substantial amount of iron [95]. This would stimulate photosymbiont growth and exacerbate the cellular processes that trigger bleaching, suggesting this trait is detrimental rather than beneficial to the coral host under thermal stress. Although previously suggested, we do not consider it a priority to test candidate bacteria with this trait, and it has not been tested to date.

### 5.6. Phosphate-Solubilising Activity

Phosphate-solubilising activity has been proposed as a beneficial trait to support coral metabolism [72]. Phosphate-solubilising bacteria are capable of releasing inorganic phosphate from insoluble compounds that are typically found in soil. As far as we know, such compounds are not abundant in the coral mucus [96] and therefore the hypothesis that phosphate-solubilising activity is beneficial for mitigating coral bleaching is tenuous. Instead, bacterial phosphatase activity, which can release phosphate from organic phosphorus compounds like nucleotides, phospholipids, or sugar phosphates that are common in the dissolved organic phosphorus pool, may be a more relevant trait to explore. Bacterial phosphatase activity could play a role in internal phosphorus cycling, potentially increasing available phosphate for other holobiont members [97].

### 5.7. Urease Activity

Urease activity may enhance coral calcification [72,92]. Urease catalyses the hydrolysis of urea into CO_2_ and ammonium [98]. On coral reefs, urea is a dynamic and sometimes abundant source of bioavailable nitrogen [99]; thus bacterial urease activity [100] can in theory provide ammonium to the coral holobiont and boost its metabolic processes such as calcification [101]. However, under thermal stress, the additional ammonium generated by urease activity may stimulate photosymbiont growth, which can have negative consequences for the nutritional state of the coral host and bleaching, as explained below. This trait has only been suggested and remains untested in experimental bacterial manipulation approaches.

## 6. The Challenges of Enhancing Coral Thermal Tolerance Long-Term at Large Spatial Scales

### 6.1. Complexity of the Thermal Tolerance Trait

Thermal tolerance is a complex trait and, in corals, it is underpinned by many host genes of small effect size [102]. In addition, thermal tolerance of corals is strongly affected by their photosymbiont community composition [103], as well as by historical temperature regimes [104] and environmental conditions in the recent past [105]. Therefore, developing a probiotic that enhances thermal bleaching tolerance is more complex compared to, for instance, the development of a probiotic aimed at mitigating pathogen-driven diseases such as stony coral tissue loss disease (SCTLD) [106] or coral bleaching induced by certain *Vibrio* bacteria [107]. Mitigating disease requires the probiotic bacterium to have an antagonistic effect against the pathogen, which can be relatively easily tested in the laboratory. However, the bacterial traits required to abate coral bleaching are less apparent. There are several theories of coral bleaching, each invoking different cellular processes (Figure 2a). The first hypothesis (indicated by the number 1 in a red circle in Figure 2a) postulates that elevated temperatures and light intensities can damage the photosynthetic apparatus of the photosymbionts, impairing its ability to process light photons and resulting in an increased production of ROS and possibly reactive nitrogen species (RNS). H_2_O_2_ produced by the photosymbionts can diffuse into the coral host cell, where it may be decomposed to O_2_ by host catalases (‘CAT’ in Figure 2a). Within the host cell, O_2_ and O_2_-sensitising metabolites (‘Sens.’) released by photosymbionts may react to form singlet oxygen (^1^O_2_). O_2_^−^ can react with nitric oxide (NO), to form peroxynitrite (ONOO^−^), which inhibits mitochondrial electron transport. Within coral mitochondria, O_2_^−^ production increases due to elevated respiration or electron transport inhibition. Endoplasmic reticulum (‘ER’) stress can result in the release of calcium (Ca^2+^) to the mitochondria. Increased protein folding and repair demand within the ER further contributes to ROS production. When the host antioxidant systems are overwhelmed, increased RNS, ROS or mitochondrial Ca^2+^ uptake disables the mitochondrial B-cell lymphoma 2 (BCL2) protein, which normally suppresses apoptosis by inhibiting the release of cytochrome *c* (Cyt *c*). Cyt *c* released into the cytoplasm binds to the apoptosome, triggering caspase activation and ultimately leading to host cell apoptosis (including photosymbiont-containing host cells) [76]. The second theorem (indicated by the number 2 in a blue circle in Figure 2a) states that dealing with thermal stress is energy-demanding, making it difficult for the coral host to maintain its energetically costly carbon-concentration machinery. This can result in a failure of the coral animal to provide a sufficient supply of CO_2_ to its photosymbiont partners, particularly during periods of high solar radiation when the photosynthetic demand for CO_2_ is maximal (e.g., during summer heatwaves when cloud cover and wind are typically low) [108]. The ensuing disruption of the Calvin-Benson cycle and electron transport chain within the photosymbiont chloroplasts leads to an increased production of ROS, which leak into the coral cells where they cause symbiont loss. The third theory (indicated by the number 3 in a green circle in Figure 2a) postulates, the high energetic cost of heat stress triggers a shift from anabolic to catabolic processes within the coral host, with catabolism causing the release of ammonium. One of the mechanisms by which the coral host controls photosymbiont growth under non-stress conditions is by limiting the amount of nitrogen the photosymbionts can access. However, the high amount of catabolically produced ammonium liberates the photosymbionts from this nitrogen restriction, allowing them to use most of the photosynthetically fixed carbon for their own growth instead of translocating it to the host [78]. As a result, the host becomes even more nutrient-starved, and the fast-growing photosymbionts run out of phosphorus. This nitrogen:phosphorus imbalance alters the lipid composition and reduces functionality of the chloroplast thylakoid membranes that house the photosystems [109], potentially making them thermally unstable [110]. Again, the ultimate outcome is an excess production of ROS that leak into the host cells and trigger the loss of photosymbionts.

These three interlinked bleaching theories suggest that bacterial traits such as ROS-scavenging and nutrient/energy provisioning may assist corals to cope with heat stress, which could be relevant at different stages of the bleaching cascade (Figure 2a). This notion is supported by the observation that relative to thermosensitive colonies, thermally tolerant coral colonies tend to show constitutively higher expression levels of a number of genes, including those encoding antioxidant synthesis, as well as others such as heat shock proteins, the unfolded protein response, the cytoskeleton and calcium signalling [111]. Further, corals with better energy reserves are generally more tolerant to heat stress [87]. The nutrient-deprived state of the coral host may be ameliorated by other bacteria and traits, such as carbon fixation and export (Figure 2a). The beneficial bacterial traits proposed here and in the literature are currently speculative, and it is critical for progressing the field to obtain a better understanding of the mechanisms by which candidate bacteria enhance coral thermal tolerance, so that a more targeted candidate selection strategy can be developed.

### 6.2. Lack of Mechanistic Understanding of Improved Coral Thermal Resilience Resulting from Bacterial Administration

In other invertebrate hosts, microbial mechanisms responsible for enhancing thermal tolerance include the stimulation of expression of host thermal response genes, the direct action of metabolites and proteins produced by candidate bacteria on host health, or metabolites that may be released to the host upon heat-induced lysis of microbial cells [112]. Further, bacterial administration may alter the composition and function of the resident coral microbiome [58,113]. As discussed above, laboratory studies have demonstrated that inoculation of adult corals with probiotic candidate bacteria can enhance coral thermal resilience for short periods of time, but the mechanisms behind this enhancement are largely unknown, and it remains unclear whether long-term resistance can be achieved. Few coral phenotypic traits are typically assessed during experimentation with probiotic bacteria and, to date, bacterial traits within the holobiont have not been examined, which has resulted in a poor understanding of how the candidate bacteria enhance coral thermal tolerance. In only one study [66] was the impact of inoculating coral with a bacterial consortium on the holobiont phenotype examined in conjunction with host gene and metabolite expression in response to heat stress. Inoculated corals recovered better from heat stress and showed lower dimethylsulfoniopropionate (DMSP) concentrations, which correlated with DMSP degradation genes present in one of the administered bacteria. Maintenance of coral lipid concentrations and upregulation of lipid biosynthesis genes, as well as upregulation of thermal stress protection genes and downregulation of apoptosis genes in inoculated corals, may also have contributed to their higher thermal resilience. However, the bacterial strain and its specific traits that were responsible for the host transcriptomic changes remain to be resolved. Future studies should also examine the bacterial candidate’s transcriptomic response so that bacterial traits responsible for host phenotypic enhancements can be deciphered. Insight into how candidate bacteria enhance coral thermal tolerance is essential to refine strategies for selecting candidates, and this will accelerate the development of bacterial manipulation methods in different reef settings.

The use of different bacterial strain combinations, often tested in consortia rather than individually (Table 1), makes it difficult to compare outcomes and identify which candidates are most effective. Genera commonly used include *Pseudoalteromonas*, *Halomonas*, *Cobetia*, *Bacillus*, and *Roseobacter*. Because these were applied in mixed consortia, it is unclear which genera or strains drive observed effects within the coral holobiont. Studies testing individual strains, such as in Dungan et al. [114], provide more insight into strain-specific contributions to cnidarian thermal tolerance. Although this study found no differences between strains under heat stress, likely due to bacterial loss from the host before thermal stress exposure, it incorporated both ROS-scavenging and non-ROS-scavenging strains (as demonstrated by phenotypic assays), enabling more informative comparisons.

Further, the only negative control typically used is a no-inoculum control, which does not assess the effect of inoculation with live microorganisms per se (even if they lack putative beneficial traits), such as inducing changes in the resident microbiome composition. There is also the possibility that bacterial inocula serve as a food source, which may be particularly relevant when corals in laboratory experiments are not given other feeds and bacterial inoculations are frequently applied. Indeed, corals that feed heterotrophically have been shown to recover better from thermal bleaching [115]. Assuming the biomass of a bacterium is one picogram (10^−12^ g), the wet-to-dry weight is 50% [116], the mean ratio of ash-free dry weight to dry weight is 80% [117], and the mean calorific value of microorganisms is 5.4 kcal.g^−1^ ash-free dry weight [118], the calorific value of a single bacterium is ~2.15 × 10^−9^ cal. Bacterial inoculations in coral studies are typically carried out with 10^8^–10^10^ bacteria, which equates to <1 to ~120 calories, but note that it is unknown which portion of the inocula is consumed. For comparison, newly hatched brine shrimp nauplii (*Artemia salina*), which are commonly used as coral feed, have a calorific value of 9.24 × 10^−3^ cal per freshly hatched individual [119]. At densities of 1–4 nauplii per ml of seawater, *Pocillopora acuta* fragments of ~153 cm^2^ capture ~646–1507 nauplii per hour [120], which represent a calorific value of ~5.97–13.92 cal. If bacterial inocula are consumed, their calorific value is thus of a similar order of magnitude as that of brine shrimp nauplii at densities commonly used in coral aquaculture, and they may therefore have beneficial effects on coral thermal resilience simply due to their nutritional value.

### 6.3. Ability of Candidate Bacteria to Persist Within the Coral Microbiome

Long-term persistence of probiotic bacteria (i.e., the permanent establishment of these bacteria within the coral holobiont) is essential for sustained beneficial effects such as enhanced thermal tolerance, particularly for restoration applications where repeated inoculations over hectares of reef surface are impractical or costly [121]. Whether administered candidate bacteria can remain associated with recipient corals is still elusive, either because this was not assessed in studies to date, the experiments were run for a relatively short period of time, the short region of the 16S rRNA gene used for metabarcoding could not distinguish between candidate bacteria and closely related resident bacteria, and/or multiple inoculations were carried out throughout the experiments. Some studies report the detection of candidate bacteria shortly after their last inoculation via 16S rRNA gene metabarcoding [72], but this does not necessarily indicate integration of living candidate bacteria into the resident microbiome because bacteria (or their DNA) can be physically present in corals within the first few days post-inoculation even if they are no longer viable. For instance, heat-killed *E. coli* can be detected in recipient corals for at least six days after the last inoculation [74].

The only compelling evidence of a non-pathogenic bacterium truly having been incorporated into the coral microbiome following inoculation is a study where the bacterium *Endozoicomonas acroporae* was administered to recently settled coral recruits. Using fluorescence in situ hybridisation (FISH) with an *Endozoicomonas*-specific probe, *E. acroporae* was visualised as aggregates within the coral tissues one and six days after the last inoculation, while the genus-specific FISH probe did not reveal any signal in the control corals [74]. Early life stages have not yet developed a mature microbiome and there is likely more availability of ecological niches that may permit a probiotic bacterium to establish relative to adult corals. Cardoso et al. [67] used FISH to localise the genera of candidate bacteria in adult corals but found positive hybridisation for the candidate genera *Halomonas* and *Cobetia* in both no-inoculum control and inoculated samples (albeit in different locations within the coral tissues), indicating closely related bacteria were already present as part of the coral’s resident microbiome.

## 7. Moving the Field Forward

To accelerate the advancement of bacterial manipulation for enhancing long-term thermal resilience of corals at spatial scales that are ecologically relevant, we propose a series of improvements for future experiments below.

### 7.1. Bacterial Candidate Selection

We recommend isolating candidates from coral hosts, as seawater-derived bacteria may lack host-adapted traits required for stable associations and functional integration. Ideally, strains should originate from coral tissues or *in hospite* photosymbionts. Tissue-associated bacteria are often part of the stable core microbiome [45] and are therefore more likely to provide persistent benefits. In addition, photosymbiont-associated bacteria [43,122] may directly neutralise ROS generated by the photosymbionts. Strain selection can be guided by microscopy (localisation) and/or sequencing data from coral tissues or coral-derived algal cultures. Examples of potentially promising genera include *Endozoicomonas*, *Ruegeria* (tissue-associated strains identified) and *Roseibium*, *Marinobacter* or *Roseovarius* (strains associated with the photosymbionts).

Candidate strains should be evaluated for traits relevant to bleaching mitigation, such as alleviation of oxidative stress and carbon provisioning. This may include genomic evidence of the presence of relevant pathways and genes, followed by phenotypic validation of certain activities by candidate bacteria in culture. Once the ability of numerous candidates to enhance coral thermal tolerance has been assessed, a comparison of genomic and/or transcriptomic traits of thermotolerance-conferring versus non-conferring bacteria may permit the identification of marker genes or in vitro phenotypes for selecting additional candidate bacteria. Such adaptive method development will accelerate the adoption of this intervention at other reef locations around the world.

Additional traits should also be explored. For instance, bacterial phosphatase-mediated provisioning of phosphorus might prevent destabilisation of the thylakoid membranes and reduce ROS production during thermal stress. Phosphocholine (PC) lipids (synthesised by the coral host) are also important and occur at lower concentrations in thermally sensitive corals [123,124]. Bacteria able to produce precursors that facilitate the synthesis of these compounds may therefore represent good candidates. Further, recent metabolomic studies point to an important role for the abundance of diacylglyceryl-3-O-carboxy-(hydroxymethyl)-choline (DGCC) betaine lipids (synthesised by the photosymbionts)—lower levels of DGCC correlate with higher sensitivity to thermal bleaching [123,124,125].

To ensure environmental safety, genomes and plasmids of candidate strains should be screened for virulence factors, toxin genes, and mobile antibiotic resistance elements. Genera with a history of pathogenicity in marine environments, such as *Vibrio* spp., should be treated with caution or avoided altogether.

Long-term persistence of the candidates within the coral microbiome is essential for building sustained thermal resilience of the coral host, particularly for reef restoration applications where repeated inoculations are logistically challenging or costly. Strain selection should consider genomic features associated with host colonisation and persistence, such as ankyrin and WD40 motifs [126,127]. Further, probiotic bacteria persist longer in the human gut if the resident microbiome is underrepresented in genes harboured by the probiotic strain [128], and this could also be considered in coral experiments. A reduction in the diversity and/or abundance of the microbiome, e.g., by antibiotic treatment [129] or exposure to sterile seawater [130], prior to inoculation should be explored in corals. Autochthonous (more commonly referred to as “homologous” in the coral literature) bacteria seem to establish better in the human gut compared with allochthonous (heterologous) bacteria, and again, this could be tested in corals. We anticipate that for coral reef restoration there will be a need to inoculate both early life stages and adult colonies depending on the reef location and infrastructure available. However, the evidence to date (albeit limited) suggests stable incorporation of administered bacteria is more likely when early life stages are inoculated [74,131], possibly because more unoccupied niches are available for new bacterial symbionts to establish. Experimental validation of long-term colonisation and viability within the host is required [121], ideally over extended pre- and post-thermal stress periods (months), and evidence of persistence across coral life stages or potential for transmission across life stages from the literature should be obtained. The location of candidate bacteria within the holobiont should also be visualised, using confocal microscopy combined with taxon-specific fluorescent 16S rRNA probes (FISH), a set of strain-specific probes spanning the entire genome (GenomeFISH [132]), or pre-labelling candidates with fluorescent dyes or stable isotopes prior to administration, to determine whether localisation is important for providing benefits to the coral holobiont (e.g., do ROS scavengers need to co-localise with the photosymbionts?).

Finally, candidates with broad ecological distributions, found across diverse coral species, life stages, and regions, may have greater potential for generalisable application. Genera such as *Endozoicomonas*, *Ruegeria*, members of the *Rhodobacteriaceae*, and certain Cyanobacteria exemplify taxa with broad ecological presence.

### 7.2. Experimental Design

The number and timing of inoculations with candidate bacteria must be aligned with restoration practices. For example, if sexually produced early life stages of corals are used, inoculations should occur over the time that the corals are in the conservation aquaculture phase (typically one week over which one or a few inoculations could be carried out) or during transportation of larval slicks from the source to recipient field location [133]. At least initially, the use of consortia of multiple bacterial strains should be avoided until the impact of individual strains is well understood and a mechanistic appreciation has been obtained. At a later stage, combinations of strains can be tested. However, it is important to first assess potential antagonistic or synergistic interactions between strains to ensure that beneficial members are not outcompeted within the consortium. In addition to a no-inoculum control, a live bacteria control (lacking the selected candidate functions) and/or heat-killed bacteria need to be included to test for the possibility that the administered bacteria may impact the host phenotypes without having target traits, for instance by altering the resident microbiome or by serving as food.

An additional factor that should be considered is coral host genotype, as it may influence bacterial uptake and persistence. Studies adding different Symbiodiniaceae strains to chemically bleached corals have already demonstrated that coral host genotype can strongly affect the success of symbiont establishment, with considerable variability observed across genotypes [134]. When manipulating coral bacterial microbiomes, most studies have randomised host genotype, which is a valid experimental approach but limits our understanding of whether bacterial persistence can be achieved across genotypes. Early studies with replication across treated coral genotypes have shown that incorporation of some bacterial candidate strains can indeed vary between coral genotypes [135]. Similarly, in the coral model, the sea anemone *Exaiptasia diaphana*, different host genotypes have been shown to affect bacterial colonisation success [114].

### 7.3. Host and Bacterial Candidate Traits Assessed

The coral responses to inoculation need to be studied in more detail as this will help gain mechanistic understanding of how the candidate bacteria enhance coral thermal tolerance. Often, holobiont traits are limited to photosynthetic performance and coral colour as a categorical proxy for quantitative measures of photosymbiont density. Better alternatives are to either conduct photosymbiont cell counts, or—if insufficient biomass is available—to use quantitative PCR on the DNA extracts that are also used for metabarcoding to determine photosymbiont to coral host cell ratio [136]. Other holobiont phenotypes relevant to the traits of the candidate bacteria must also be assessed, such as energy reserves and ROS concentration within the photosymbionts and coral tissues. Coral fitness benefits can and should be linked to the phenotypes of candidate bacteria via methods such as metatranscriptomics (RNA sequence analysis of eukaryotic and prokaryotic members of the holobiont) or single-cell RNA sequencing [137]. In this manner, bacterial gene expression analyses conducted post-inoculation under thermal stress can confirm or reject *in hospite* expression of key bacterial traits (e.g., catalases, *dmdA*) and directly link candidate gene activity to observed coral responses. From such iterative analyses, more refined criteria for candidate selection may be developed. Additionally, characterisation of bacterial exudates will provide better insight into how they affect coral thermal tolerance. For instance, in vitro validation of glucose export via semiSWEET transporters using stable isotope labelled glucose assays can be employed to assess whether bacteria provide carbohydrates to the coral host.

### 7.4. Moving from Laboratory to Field Experiments

A common pathway for the safe implementation of reef interventions is to obtain proof-of-concept and some understanding of ecological risks through laboratory experimentation prior to the commencement of small field trials [138]. Despite a current lack of mechanistic understanding, thermal enhancement of corals following bacterial administration has indeed been demonstrated in the lab, providing proof-of-concept of this intervention. Further, the only field inoculation study aimed at enhancing coral thermal tolerance so far (which was conducted in the Red Sea) reported no negative impacts on the surrounding biota and the inoculants were not detected in surrounding water and sediments despite 36 inoculations having been carried out [72]. In another study, a probiotic bacterium to control SCTLD was tested on healthy specimens of five non-target coral species held in aquaria, with no declines in coral health observed 21 days following inoculation. This bacterium was subsequently administered to corals in the field, and no adverse effects on the surroundings were reported in this study [106]. There will be great value in fast-tracking our ability to move bacterial manipulation experiments from the laboratory to the field in other parts of the world. The natural environment is far more complex than laboratory settings, and laboratory findings always require field validation. A way to assess whether reef-scale thermal enhancement has been achieved is to survey the performance of inoculated corals during summer heatwaves and those that have not been inoculated with probiotic bacteria across multiple reefs. We encourage researchers to conduct risk assessments for field experiments, where risks could be minimised by using bacteria that have been previously isolated and cultured from local or at least regional corals. When using coral early life stages, inoculations can be restricted to the conservation aquaculture stage. Adult corals can be removed from the field, inoculated in the laboratory, and then returned to the field, although this is labour-intensive when large numbers of colonies need to be inoculated. Alternatively, inoculations can be conducted in the field but carried out over a short period of time (e.g., days). For such field inoculations, the candidate bacteria can be injected into a weighted bag placed over a coral colony, as was successfully done for the treatment of SCTLD [106] or can be released into the water immediately above the coral colony [72]. It will of course be critical to obtain Free and Prior Informed Consent (FPIC) from relevant Traditional Owner groups or other local communities, and approval from the regulators prior to commencing field trials.

### 7.5. Methodological Improvements

Absolute abundances of bacteria are rarely assessed in coral probiotic studies [65], and changes in relative abundance are often tacitly interpreted as changes in absolute abundances. Quantitative PCR assays should be included in future studies to assess bacterial load and changes therein as well as changes in absolute abundance of inoculants and members of the resident microbiome. Further, corals heterotrophically feed on bacteria; because standard metabarcoding assays do not distinguish between live and dead bacteria or free bacterial DNA, these assays can cause errors in the inferred microbiome composition. Going forward, methods to specifically characterise the living or metabolically active bacterial community (e.g., by treating samples with propidium monoazide (PMA) prior to metabarcoding [139], or using RNA-based metabarcoding) should be used.

Finding the inoculants within the coral microbiome is often based on sequence identity in a short region of the 16S rRNA gene and genus-level assignment. This approach cannot distinguish among closely related species and often the inoculant has an identical sequence to one or several resident strains. To more reliably assess whether candidate bacteria remain with the recipient coral, long-read metabarcoding technologies that can cover a large portion of the 16S gene, such as Oxford Nanopore Technologies and Pacific Biosciences, provide a far better choice. Similarly, the specificity of FISH to localise inoculants can be improved by using GenomeFISH [132], which applies many probes distributed across the target genome rather than traditional FISH approaches that typically rely on a single taxon-specific probe. In this manner, unintended probe binding to closely related resident strains can be minimised.

## 8. Closing Remarks

Bacterial manipulation of corals is an emerging field, particularly where it is aimed at enhancing thermal bleaching tolerance. While limited, published findings are promising in that enhanced tolerance has been recorded in laboratory experiments. However, the gap to implementing this intervention as part of coral reef restoration initiatives remains enormous. In this article, we recommend several changes to experimental designs and methods typically used. We are in a race against time to ensure the persistence of corals and coral reefs until the climate has stabilised, with extreme summer heatwaves of >20 degree heating weeks (DHW) already having occurred in Florida [140] and north-west Australia [7] in the past 2–3 years. Coral bleaching generally occurs at 4 DHW with severe bleaching and mortality expected at 8 DHW [141]. Sufficient enhancement of thermal resilience of corals likely requires the use of multiple intervention approaches, including bacterial and photosymbiont manipulation [103,138] and selective breeding [142], as well as a reduction in bleaching stressors via engineering interventions such as shading and cooling [143]. Therefore, optimising experimental methodologies to accelerate the advancement of individual interventions is critically important.

## Data Availability

No new data were created or analyzed in this study. Data sharing is not applicable to this article.
